# Clinically uncomplicated *Plasmodium falciparum *malaria with high schizontaemia: A case report

**DOI:** 10.1186/1475-2875-7-57

**Published:** 2008-04-11

**Authors:** Khin Maung Lwin, Elizabeth A Ashley, Stephane Proux, Kamolrat Silamut, François Nosten, Rose McGready

**Affiliations:** 1 Shoklo Malaria Research Unit, Mae Sot, Tak, 63110, Thailand; 2 Mahidol-Oxford Tropical Medicine Research Unit (MORU), Faculty of Tropical Medicine, Mahidol University, Bangkok, Thailand; 3 Centre for Clinical Vaccinology and Tropical Medicine, Headington, Oxford, UK

## Abstract

**Background:**

The treatment options for acute *Plasmodium falciparum *malaria are based on the clinician classifying the patient as uncomplicated or severe according to the clinical and parasitological findings. This process is not always straightforward.

**Case presentation:**

An adult male presented to a clinic on the western border of Thailand with a physical examination and *P. falciparum *trophozoite count (1.2% of infected red blood cells, IRBC) from malaria blood smear, consistent with a diagnosis of uncomplicated *P. falciparum *infection. However, the physician on duty treated the patient for severe malaria based on the reported *P. falciparum *schizont count, which was very high (0.3% IRBC), noticeably in relation to the trophozoite count and schizont:trophozoite ratio 0.25:1. On intravenous artesunate, the patient deteriorated clinically in the first 24 hours. The trophozoite count increased from 1.2% IRBC at baseline to 20.5% IRBC 18 hours following the start of treatment. By day three, the patient recovered and was discharged on day seven having completed a seven-day treatment with artesunate and mefloquine.

**Conclusion:**

The malaria blood smear provides only a guide to the overall parasite biomass in the body, due to the ability of *P. falciparum *to sequester in the microvasculature. In severe malaria, high schizont counts are associated with worse prognosis. In low transmission areas or in non-immune travelers the presence of schizonts in the peripheral circulation is an indication for close patient supervision. In this case, an unusually high schizont count in a clinically uncomplicated patient was indicative of potential deterioration. Prompt treatment with intravenous artesunate is likely to have been responsible for the good clinical outcome in this case.

## Background

The 2006 WHO malaria treatment guidelines [1] laid out in a user friendly volume give treatment recommendations based on the available evidence. The severity of malaria, and hence treatment options, are determined from the clinical features of a patient and supported by the laboratory detection of parasites in the blood. There is a variable relationship between parasite density and disease severity, as recognized by Field [2] and in general the higher the parasite count, the more severe the infection. However, this is not a linear relationship. High parasite counts can be found in asymptomatic individuals [3], while some others can die of cerebral malaria with no parasites detected in the peripheral blood, although this is rare and was only observed in the largest series of cerebral malaria autopsies following effective antimalarial treatment [4]. On the Thai-Burmese border, patients with more than 4% IRBC, but no clinical signs of severe infection had a case fatality rate of 3% compared to an overall case fatality of 1.9 per 1000 for malaria [5] i.e. 15 times higher. Beside the absolute parasite density, the presence of schizonts on the blood film and pigment in the neutrophils are good indicators of more severe infection and warrant the clinician's attention as these patients may rapidly deteriorate. The following case demonstrates another of the vagaries of malaria diagnosis, which presented a management dilemma beyond the scope of the current WHO malaria treatment guidelines.

## Case presentation

A 28 year old male from the eastern border of Burma walked to a malaria clinic on the Thai side of the river that marks much of the border between the two countries. He complained of a three-day history of fever, nausea, headache and dizziness. He denied ever having had malaria in the past. He was fully conscious, able to answer all questions appropriately and had an aural temperature of 37.5°C, pulse 88 beats/min, respiratory rate 20/min and blood pressure 110/60 mmHg. His liver could just be palpated, 1 cm below the costal margin in the mid-clavicular line and his spleen was not palpable. Examination was otherwise unremarkable. In particular, he had no signs suggestive of severe malaria [6].

**Figure 1:**
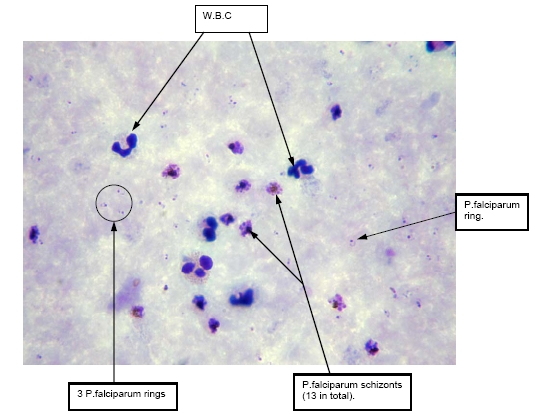
Thick blood film of patient on admission demonstrating significant presence of schizonts compared with trophozoites (oil immersion × 1000).

At the outpatient department, a rapid diagnostic test (Paracheck^®^Pf) result was strongly positive and, consequently, a malaria blood smear was taken. The malaria smear result (12:20 hrs) was *Plasmodium falciparum *trophozoites 12/1,000 RBC (equivalent to a parasitaemia of 51,245/μL (or 1.2% IRBC). *Plasmodium falciparum *schizonts were present on the slide and the count was high: 3/1,000 RBC (equivalent to a schizontaemia of 12,811/μL or 0.3% IRBC). *Plasmodium vivax *trophozoites were also noted at low density: 8/500 White Blood Cells (WBC) (or a parasitaemia of 128/μL). Scarce malaria pigment was observed in < 1% of polymorphonuclear leucocytes (neutrophils) (Figure 1). On admission his capillary blood haematocrit (HCT) was 34% and blood glucose 102 mg/dL.

The patient exhibited no signs of severe malaria but was admitted to hospital and treated according to the WHO severe malaria protocol [1] on the basis of the high schizont count (Table 1) and the presence of pigment. He received a 5% dextrose infusion with 2.4 mg/kg IV artesunate (currently the most potent antimalarial) at 0, 12 and 24 hrs, then every 24 hours for a total of five doses. The available blood smear results during this patient's entire admission have been summarized (Table 1). The trophozoite parasitaemia increased from 1.2% to 20.5% in approximately 18 hours (Table 1). In the first 24 hours, the clinical condition of the patient deteriorated as he developed hyperpyrexia (temperature 40°C), associated with worsening headache and recurrent vomiting. His urine was blood-stained, but this had resolved by the 2^nd ^day of treatment. His urine output was monitored, as was blood glucose (6-hourly), and these were all just within normal limits. The patient felt very well on the 3^rd ^day of treatment but did have a spontaneous nose bleed on this day. The treatment was changed to oral artesunate and mefloquine (15 mg/kg on the 6^th ^day and 10 mg/kg on the 7^th ^day of treatment). Fever clearance time was 66 hours and time to parasite clearance (based on 24 hourly blood smears) was 144 hours. This patient had a negative thalassaemia screen, mild anaemia and thrombocytopaenia, by complete blood count (taken on the 4^th ^day of treatment): white blood cell 8.9 × 10^3^/μL, haemoglobin 11.2 g/dL and platelet count 125 × 10^3^/μL,

**Table 1:** Summary of malaria blood smear results, haematocrit and *P. falciparum *parasitaemia, until malaria smear negative.

**Time hours**	***P. falciparum *trophozoite count**	***P. falciparum *Parasitaemia/uL**	***P. falciparum *schizont count**	***P. vivax *trophozoite count**	**Malaria pigment in neutrophils**	**HCT%**
H 0	12/1000 RBC	51,245	3/1000 RBC	8/500 WBC	< 1%	34
H 18	205/1000 RBC	725,340	9/500 WBC	Neg	2+	34
H 42	88/1000 RBC	338,492	Neg	Neg	1+	-
H 66	10/500 WBC	178	Neg	Neg	1+	-
H 90	2/500 WBC	36	Neg	Neg	Neg	-
H 114	neg	-	Neg	Neg	Neg	-

## Conclusion

This man had a presenting falciparum trophozoite parasitaemia of 51,245/μL and a very high schizont parasitaemia of 15,072/μl (Table 2) (total 67,117/μl) with no clinical signs that would guide the physician towards classifying this patient as having severe malaria [3]. His parasitaemia was well below the WHO Malaria Treatment Guidelines 2006 cut-off for hyperparasitaemia (4% IRBC in a low transmission area ≈150,000/μL) and the concentration of pigment containing neutrophils (<1%) was also well below the 5% or greater concentration that has been associated with a poor prognosis [7]. The only sentence in the guidelines which might alert the physician to the potential seriousness of this case was: "At any parasitaemia, prognosis worsens if there is a predominance of more mature parasite stages [1]."

**Table 2:** Proportion of patients presenting with schizonts, mean schizontaemia/counts and schizont:trophozoite ratio, according to malaria severity.

Malaria Severity	Proportion of patients with schizonts on admission % (n)	Geometric mean [range] schizontaemia/μL	Maximum *P. falciparum *schizont count	Schziont: Trophozoite Median [range] Ratio in peripheral blood	Source
Uncomplicated	4.0 (21/530)	16 [16–65]	4/500 WBC	0.0003:1 [0.00008–0.026]	[14]
	3.0 (15/499)	29 [16–158]	10/500 WBC	0.0013:1 [0.00008–0.12]	[6]
	5.0 (25/500)	24 [16–129]	8/500 WBC	0.00087:1 [0.000055–0.0684]	[15]
Hyper-parasitaemic >4% RBC parasitized	20.9 (124/594)	31 [16–324]	20/500 WBC	0.00007:1 [0.000013–0.00131]	EA. Ashley#
Severe	20.3 (196/966)	6,685 [5–66,558]	17/1000 RBC*	0.09:1 [0.01 – 4.88]	[16]
Case report patient	n.a.	12,811	3/1000 RBC	0.25:1	-

RBC = red blood cell; WBC = white blood cell; n.a. = not applicable

* This patient died

**Source**: [14–16]

# personal communication

Table 2 summarizes schizont count results and schizont: trophozoite ratio from patients from the same population, with acute uncomplicated falciparum malaria on the Thai-Burmese border, or with severe malaria from SE Asia. This patient had a schizont count in the same range as has been more commonly reported from patients presenting with hyperparasitaemic and severe malaria and a high ratio of schizonts to trophozoites. This case is a reminder to consider all the details of a malaria smear when deciding on treatment. The physician on duty was alerted by the malaria smear result despite the fact the patient was clinically uncomplicated. The trophozoite and schizont count were rechecked immediately. The decision to give severe malaria treatment was probably very wise considering the early clinical deterioration.

High parasite densities are frequently equated with severe disease but the reverse is not always true. There may be wide differences between the number of parasitized cells in the peripheral blood smear and the number sequestered in the microvasculature[3,8]. This case was unusual because it is the mature stages that are normally sequestered and as a result not picked up by peripheral blood microscopy. It is logical that a high schizont count should be considered a danger sign – one *P. falciparum *schizont releases an average of 16 merozoites, each of which can theoretically infect another RBC causing the infective biomass to expand exponentially. Modelling data of real patients with acute uncomplicated falciparum malaria, White and co-workers [9] demonstrated that during the rising phase of the infection the ratio of circulating to sequestered parasites was more dependent on synchronicity of infection than multiplication rate. As well, when the mean stage of parasite development is in the second half of the asexual life cycle, synchronous infections showed considerable fluctuations.

Although antimalarials are frequently referred to as blood schizontocides, in fact mature trophozoites are more susceptible to the drugs and formed schizonts are relatively drug- resistant [10]. Young ring trophozoites are also relatively drug resistant (particularly to quinine and pyrimethamine). Chloroquine acts principally on the large ring-form and mature trophozoite parasite stages. The main stage targeted by quinine is the mature trophozoite while the antifols act a little later [11]. The artemisinin derivatives have the broadest time window of antimalarial effect (from ring forms to early schizonts). These compounds prevent maturation of ring stages, thus reducing subsequent cytoadherence responsible for severe disease [12]. Hence an artemisinin derivative was the drug of choice in this case and the parenteral route ensured adequate drug levels were reached quickly.

This report comes from an area of low transmission. In areas of low transmission where immunity remains poor across all age groups, or in returned travelers, even a low schizont count is an indicator for close patient observation. The advantages of access to high quality microscopy with parasite staging are shown here but would be considered a luxury in many malaria endemic settings [13], where accurate parasite identification and quantification may not be possible. Increasing reliance on rapid diagnostic tests is likely to increase accuracy of malaria diagnosis overall but certain information can only be provided by slide microscopy.

When treating *P. falciparum *malaria one needs to account for all stages reported from the malaria blood smear bearing in mind it provides only a guide to the total parasite biomass. Any clinical deterioration in a patient on oral treatment necessitates a change to the severe malaria protocol. High parasite counts, as anticipated in this patient, are best treated with artemisinin derivatives.

## Authors' contributions

KML was responsible for the treatment of the patient and consulted RM and FN, SP confirmed the blood smear findings and provided photographic evidence of the diagnosis, EAA and KS reviewed and verified data on schizonts in large treatment trials; RM, EAA, and FN and coordinated the manuscript. All authors read and approved the final manuscript.
